# Supplementary material for: Using theory of change to better address social and economic needs in mental health services
Source: Int J Ment Health Syst. 2026 Apr 24;20:5. doi: 10.1186/s13033-025-00693-7 (PMC13107605; doi:10.1186/s13033-025-00693-7)

**Supplementary Figure 1. A theory of change framework detailing how to bring social and economic inclusion to the forefront of mental health services in the catchment area.**

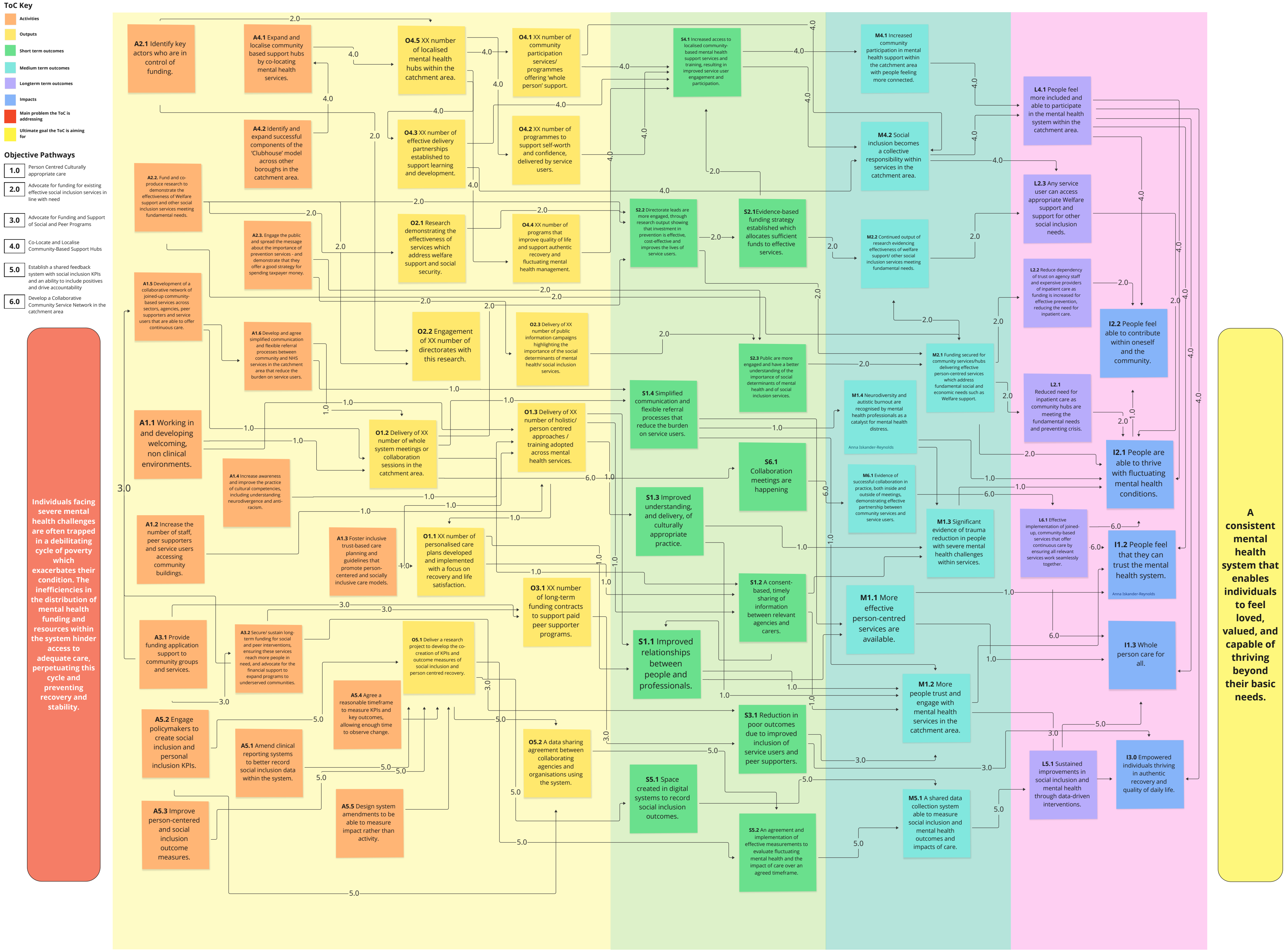

Supplement: Supplementary file 1 — Supplementary Material 1 [file 13033_2025_693_MOESM1_ESM.pdf]
